# First molecular detection of *Sarcocystis arctica* (Apicomplexa: Sarcocystidae) infecting crab-eating raccoon (*Procyon cancrivorus)* in Brazil

**DOI:** 10.1590/S1984-29612025038

**Published:** 2025-06-02

**Authors:** Julia Somavilla Lignon, Diego Moscarelli Pinto, Silvia Gonzalez Monteiro, Kauê Rodriguez Martins, Rodrigo Casquero Cunha, Diago Dutra Lima, Luíse Nunes Bonneau de Albuquerque, Camila Gonçalves da Silveira, Stanrley Victor Nascimento da Silva, Oluwagbemiga Ademola Dada, Matthew Ajani Ayoola, Felipe Geraldo Pappen, Fábio Raphael Pascoti Bruhn

**Affiliations:** 1 Laboratório de Epidemiologia Veterinária, Departamento de Veterinária Preventiva, Universidade Federal de Pelotas – UFPel, Pelotas, RS, Brasil; 2 Laboratório do Grupo de Estudos em Enfermidades Parasitárias, Departamento de Veterinária Preventiva, Universidade Federal de Pelotas – UFPel, Pelotas, RS, Brasil; 3 Laboratório de Parasitologia Veterinária, Departamento de Microbiologia e Parasitologia, Universidade Federal de Santa Maria – UFSM, Santa Maria, RS, Brasil; 4 Laboratório de Biologia Molecular Veterinária, Departamento de Veterinária Preventiva, Universidade Federal de Pelotas – UFPel, Pelotas, RS, Brasil; 5 Adeyemi Federal University of Education, Ondo, Nigeria

**Keywords:** Muscle infection, Sarcocystosis, Southern raccoon, Wild carnivore, Infecção muscular, Sarcocistose, Guaxinim do Sul, Carnívoro silvestre

## Abstract

The crab-eating raccoon (*Procyon cancrivorus*) is a wild carnivore with a broad geographic distribution, randing from Costa Rica to South America. This species remains understudied, particularly regarding *Sarcocystis* spp. infections. This study aimed to report the first molecular detection of *Sarcocystis arctica* in *P. cancrivorus*. The roadkill specimen, recovered from the highway of Pedro Osório, Rio Grande do Sul, Brazil, was subjected to necropsy. Tissues samples, bone marrow and blood were collected, and their genomic DNA was extracted. PCR amplification targeting 18S rRNA, COX1 and 28S genes, genetic sequencing and phylogenetic analysis confirmed the presence of *S. arctica* DNA in cardiac muscle samples. Molecular characterization showed 98.62-99.6% identity to sequences of this species deposited in GenBank. We report the first documentation of *S. arctica* infection in a *P. cancrivorus* heart sample. While species within the genus *Procyon* serve as definitive and intermediate hosts for other *Sarcocystis* species, it is uncertain whether *P. cancrivorus* acts as an aberrant host or plays a regular role in the protozoan life cycle, particularly in muscle tissue. Additionally, its impact on *P. cancrivorus* populations is still unknown, highlighting the need for further studies.

## Resumo

Mão-pelada (*Procyon cancrivorus*) é um carnívoro selvagem com ampla distribuição geográfica, abrangendo a Costa Rica até a América do Sul. Essa espécie é pouco estudada, particularmente em relação às infecções por *Sarcocystis* spp. Este estudo objetivou reportar a primeira detecção molecular de *Sarcocystis arctica* em *P. cancrivorus*. Um espécime atropelado, recuperado das rodovias de Pedro Osório, Rio Grande do Sul, Brasil, foi submetido à necropsia. Fragmentos de tecidos, medula óssea e sangue foram coletados e seu DNA genômico foi extraído. A amplificação da PCR, direcionada aos genes 18S rRNA, COX1 e 28S, sequenciamento genético e análise filogenética confirmaram a presença de DNA de *S. arctica* em amostras de músculo cardíaco. A caracterização molecular mostrou 98,62-99,6% de identidade com sequências dessa espécie depositadas no GenBank. Foi relatada a primeira documentação de infecção por *S. arctica* em amostra de coração de *P. cancrivorus*. Embora espécies, dentro do gênero *Procyon,* sirvam como hospedeiros definitivos e intermediários para outras espécies de *Sarcocystis*, é incerto se *P. cancrivorus* atua como um hospedeiro aberrante ou desempenha um papel regular no ciclo de vida do protozoário, particularmente no tecido muscular. Além disso, seu impacto nas populações de *P. cancrivorus* ainda é desconhecido, destacando a necessidade de mais estudos.

*Procyon cancrivorus* is a nocturnal and crepuscular wild carnivore that inhabits shrubby areas near water sources. This species is commonly known as the crab-eating raccoon, “mão-pelada” or “guaxinim-sulamericano” and has a wide geographic distribution from Costa Rica to southern South America (Wilson & Mittermeier, 2009). The genus *Procyon* also includes two other species: *P. lotor,* which ranges from southeastern Canada to the southern United States, extends through Central America to central-western and northern Panamá, and is also found in South America, particularly in Colombia, Ecuador, and Venezuela (Marín et al., 2012); and *P. pygmaeus* is found exclusively on Cozumel Island, Mexico (Wilson & Mittermeier, 2009).

*Procyon lotor* has previously been identified as a definitive host for *Sarcocystis cruzi*, *Sarcocystis tarandivulpes*, *Sarcocystis grueneri,* and *Sarcocystis miescheriana* (synonym of *Sarcocystis suicanis*), as well as an intermediate host for *Sarcocystis neurona*, *Sarcocystis kirkpatricki* and *Sarcocystis lutrae* (Dubey et al., 2016; Máca, 2020). However, information about *Sarcocytis* spp. infections in *P. cancrivorus* and *P. pygmaeus* is currently unavailable.

Parasites of the genus *Sarcocystis* affect various domestic and wild animals (e.g., birds, reptiles, and mammals, including humans) and are characterized by an obligate two-host life cycle, based on prey-predator or scavenging relationships (Dubey et al., 2016; Máca, 2020). Definitive hosts are infected by ingesting tissues containing sarcocysts which are present mainly in striated muscles of the intermediate host (asexual stage). Intermediate hosts become infected by ingesting sporocysts shed by definitive hosts (sexual phase) (Dubey et al., 2016). Carnivorous species serving as definitive hosts for one *Sarcocystis* species may also serve as intermediate hosts for other *Sarcocystis* species, harboring sarcocysts in the musculature (Kubo et al., 2009).

According to Dubey et al. (2016), the number of species of *Sarcocystis* is constantly increasing, and currently there are approximately 200 valid species (Máca, 2020). However, the complete life cycle, including identifying definitive hosts, is not completely elucidated for most *Sarcocystis* species. Until recently, carnivores were primarily regarded as the definitive hosts of *Sarcocystis*. However, many wild carnivore species are now being identified as intermediate hosts (Dubey et al., 2016). Here we report the first molecular detection of *Sarcocystis arctica* in a *P. cancrivorus*.

A roadkill specimen of a *P. cancrivorus* adult female was recovered on the highway in the city of Pedro Osório, Rio Grande do Sul (RS), Brazil (31º52'58''S; 52º47'41''W). The sampling occurred as part of a scheduled research on trypanosomatid protozoa in wild animals in southern Brazil (Registration number: Cobalto/UFPel 5604). The carcass had preserved and unexposed viscera, without the presence of fly larvae, with an estimated time of death between one and seven hours and was transported to the laboratory of the Group of Studies in Parasitic Diseases at the Veterinary Faculty of the Federal University of Pelotas (UFPel) in Capão do Leão, RS, using an isothermal box with ice, where it was immediately subjected to necropsy. Tissue fragments (spleen, liver, kidney, heart, lung and lymph nodes), bone marrow and blood were collected. Blood was collected from the animal's cardiac chambers, and marrow was collected from the femur bone, through a cross-section of the diaphyseal region. All samples (tissue, blood and bone marrow) were stored in sterile 2 mL plastic microtubes and frozen at -20°C until molecular analyses were performed.

DNA was extracted from all tissue, bone marrow, and blood samples using commercial kits: PetNAD™ Nucleic Acid Co-Prep Kit for bone marrow and blood, and the ID Gene™ Spin Universal Extraction Kit for tissues, in accordance with the manufacturer's instructions. The quality and quantity of extracted DNA were measured using an ultraviolet light spectrophotometer (Thermo Scientific NanoDrop Lite Spectrophotometer, Waltham, Massachusetts, USA) and 1% agarose gel electrophoresis. The extracted DNA was stored at −20°C until PCR was performed. To identify the presence of *Sarcocystis* spp. DNA, a conventional PCR (cPCR) was performed, amplifying the 18S rRNA, COX1, and 28S genes using the primers described in Table 1. The cPCR was conducted in a total reaction volume of 25μL, containing 2.0 μL of target DNA (50 ng/µL), 2.0 μL of mixed deoxynucleotide triphosphates (dNTP 2.5mM), 0.5 μL of each primer (10mM), 2.5μL of 10x reaction buffer, 1.25 μL of MgCl_2_ (50 mM), 0.25 μL of Taq DNA polymerase (5U/μL), and 16.5μL of sterile ultrapure water. The amplifications were carried out in a conventional thermal cycler (Bio-Rad, Hercules, California, USA). DNA from sarcocysts of *Sarcocystis gigantea* and ultrapure water were used as positive and negative controls, respectively. Amplified products were analyzed by electrophoresis on 1.5% agarose gels stained with ethidium bromide (0.5μg/mL) and visualized under ultraviolet light. A molecular weight standard of 100bp was used (Ladder 100bp, Ludwig Biotecnologia, Porto Alegre, Rio Grande do Sul, Brazil).

**Table 1:** Oligonucleotide primers used for amplification of DNA from the 18S rRNA, COX1 and 28S genes of *Sarcocystis* spp. derived from crab-eating raccoon (*Procyon cancrivorus*) in Brazil.

Primer identification	Primer sequence (5’-3’)	Gene	Product size (bp)	cPCR conditions	References
SarcoF/	CGCAAATTACCCAATCCTGA	18S rRNA	700	Denaturation at 94^o^C for 2 minutes, followed by 35 cycles at 95^o^C for 45 seconds, 54^o^C for 30 seconds, 72^o^C for 60 seconds, final extension at 72^o^C for 10 minutes, and 4°C ∝.	Ferreira et al. (2018)
SarcoR	ATTTCTCATAAGGTGCAGGAG				
SF1/	ATGGCGTACAACAATCATAAAGAA	COX1	1100	Denaturation at 94^o^C for 2 minutes, followed by 40 cycles at 95^o^C for 60 seconds, 52^o^C for 60 seconds, 72^o^C for 120 seconds, final extension at 72^o^C for 10 minutes, and 4°C ∝.	Cerqueira-Cézar et al. (2017)
SR5	TAGGTATCATGTAACGCAATATCCAT				
KL1/	TACCCGCTGAACTTAAGC	28S	1000	Denaturation at 94^o^C for 2 minutes, followed by 40 cycles at 95^o^C for 60 seconds, 54^o^C for 60 seconds, 72^o^C for 120 seconds, final extension at 72^o^C for 10 minutes, and 4°C ∝.	Gjerde & Schulze (2014)
KL3	GACTTCATTCTACTCAGGCATA				

**Figure 1:**
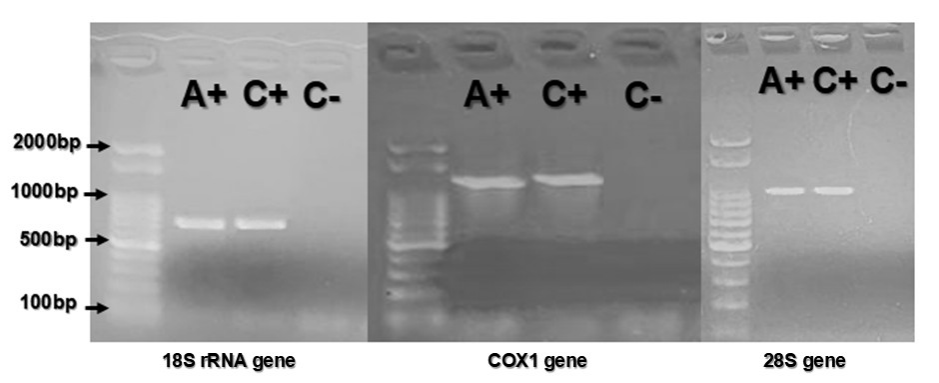
Agarose gel illustrating cPCR products for the 18S rRNA, COX1, and 28S genes. A+ represents positive sample (heart of *Procyon cancrivorus*), while C+ and C- represent positive control (sarcocysts of *Sarcocystis gigantea*) and negative control (ultrapure water), respectively.

The amplicons (Figure 1) were excised and purified using a Gel Purification Kit (Ludwig Biotechnology, Porto Alegre, Rio Grande do Sul, Brazil), following the manufacturer's recommendations, and then sequenced using the BigDye Terminator Cycle Sequencing Kit v3.1 (Thermo Fisher, USA) on an ABI3500 genetic analyzer (Applied Biosystems, USA). Consensus sequences were obtained by electrogram analysis with Phred base calling and Phrap-assembly tool, and subsequently aligned using MEGA11: Molecular Evolutionary Genetics Analysis version 11 software (Tamura et al., 2021). Multiple sequences were aligned using the ClustalW method. Sequence similarity searches with sequences deposited in the National Center for Biotechnology Information (NCBI) database were conducted using the Basic Local Alignment Search (BLAST) tool (Altschul et al., 1990). Evolutionary history for wild *Sarcocystis* species was inferred using the Maximum Likelihood method and Hasegawa-Kishino-Yano model (Hasegawa et al., 1985) for the 18S rRNA gene and using the Maximum Likelihood method and Tamura 3-parameter model (Tamura, 1992) for the COX1 and 28S genes. *Toxoplasma gondii* and *Hammondia heydorni* were used as outgroups. The MEGA11 software (Tamura et al., 2021) was also used to carry out evolutionary analyses. Statistical analysis was performed using the bootstrap method with 2000 replicates.

PCR-positive tissue samples had their tissue fragments thawed and analyzed to detect sarcocystic macrocysts, using a Leica EZ4 HD Digital Stereo Microscope (Leica Microsystems, Wetzlar, Germany). Subsequently, the fragments were macerated, mixed with 20 mL of phosphate-buffered saline (PBS, pH 7.3), and filtered through a gauze into a Petri dish. The filtrate was checked for the presence of sarcocysts microcysts, using an optical microscopy at 400x magnification, as described by Portella et al. (2021). Samples were analyzed in duplicate.

In our study, of all samples tested by cPCR, only cardiac tissue tested positive for *Sarcocystis* spp. No macrocysts or microcysts were found. Genetic sequencing identified the species as *S. arctica*.

**Figure 2:**
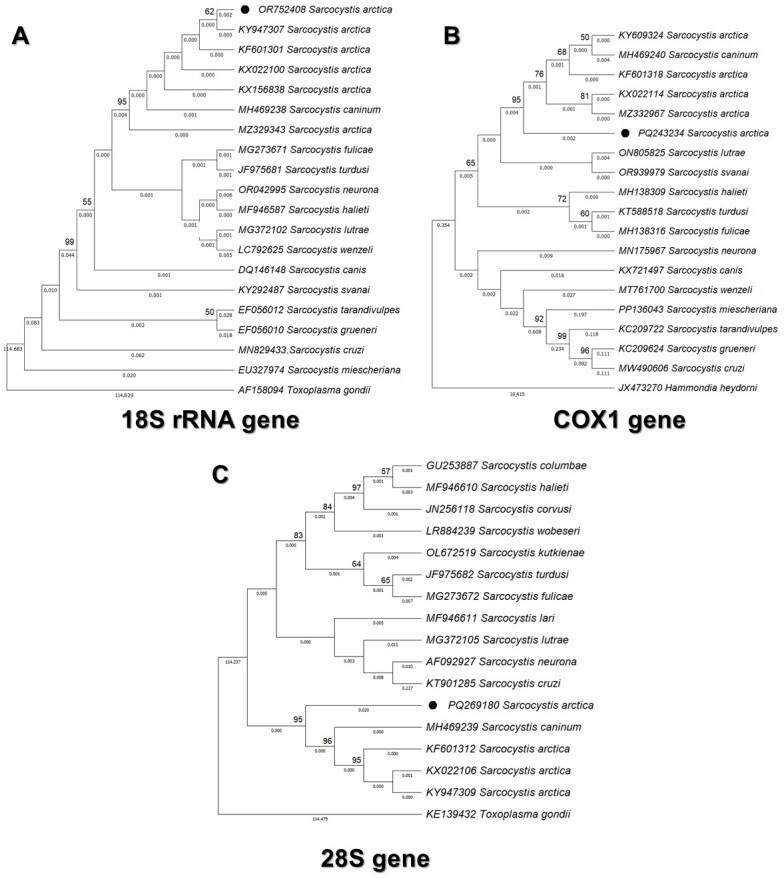
Phylogenetic trees. GenBank accession numbers for all sequences are given in front of the taxon names. Bootstrap consensus trees were inferred from 2000 replicates and probability values ​​are represented by the numbers at the nodes. A- Phylogenetic tree inferred using the Maximum Likelihood method and Hasegawa-Kishino-Yano model for wild *Sarcocystis* species based on sequences of the 18S rRNA gene. *Toxoplasma gondii* was used as an outgroup. B- Phylogenetic tree inferred using the Maximum Likelihood method and Tamura 3-parameter model for wild *Sarcocystis* species based on sequences of the COX1 gene. *Hammondia heydorni* was used as an outgroup. C- Phylogenetic tree inferred using the Maximum Likelihood method and Tamura 3-parameter model for wild *Sarcocystis* species based on sequences of the 28S gene. *Toxoplasma gondii* was used as an outgroup.

The sequences obtained for the 18S rRNA, COX1, and 28S genes of *Sarcocystis arctica* detected in the cardiac tissue sample were deposited in NCBI GenBank under accession numbers OR752408, PQ243234 and PQ269180, respectively. Our sequences showed 98.62-99.6% similarity to *S. arctica*/*S. caninum* as described in Table 2. Phylogenetic studies based on sequences of the 18S rRNA and COX1 genes placed the *P. cancrivorus* isolate in the same clade as *S. arctica*/*S. caninum* detected in red foxes (*Vulpes vulpes*) (Pavlásek & Máca, 2017), arctic foxes (*Vulpes lagopus*) from Norway (Gjerde & Schulze, 2014) and from Alaska, USA (Cerqueira-Cézar et al., 2017), Alaskan wolf (*Canis lupus*) (Calero-Bernal et al., 2016), domestic dogs (*Canis familiaris*) (Ye et al., 2018) and white-tailed eagle (*Haliaeetus albicilla*) (Máca & Gonzalez-Solis, 2022) (Figure 2). Considering the morphological and phylogenetic similarities, it has been suggested that *S. caninum* and *S. arctica* may represent the same species of *Sarcocystis* (*S. caninum* being a junior synonym of *S. arctica*) (Dubey et al., 2015; Ye et al., 2018).

**Table 2:** Similarity of our *Sarcocystis arctica* sequences obtained for the 18s rRNA, COX1 and 28S genes, compared with sequences from other authors found in the GenBank database.

GenBank accession	*Sarcocystis* species	Host	Country	Author	Percentage identity (%)	Accession length	Query cover (%)
18S rRNA gene							
KF601301	*S. arctica*	*Vulpes lagopus*	Noruega	Gjerde & Schulze (2014)	98.76	1803	100
KX022103	*S. arctica*	*Canis lupus*	USA	Calero-Bernal et al. (2016)	98.76	1194	100
KX156838	*S. arctica*	*Vulpes vulpes*	Czech Republic	Pavlásek & Máca (2017)	98.76	1517	100
KY947307	*S. arctica*	*Vulpes lagopus*	USA	Cerqueira-Cézar et al. (2017)	98.76	1545	100
MH469238	*S. caninum*	*Canis familiaris*	China	Ye et al. (2018)	98.76	1803	100
MZ329343	*S. arctica*	*Haliaeetus albicilla*	Czech Republic	Máca & Gonzalez-Solís (2022)	98.76	1772	100
COX1 gene							
KF601318	*S. arctica*	*Vulpes lagopus*	Noruega	Gjerde & Schulze (2014)	99.60	1053	100
KX022114	*S. arctica*	*Canis lupus*	USA	Calero-Bernal et al. (2016)	99.60	1021	100
KY609324	*S. arctica*	*Vulpes vulpes*	Czech Republic	Pavlásek & Máca (2017)	99.60	1012	100
MH469240	*S. caninum*	*Canis familiaris*	China	Ye et al. (2018)	99.60	1085	100
MZ332967	*S. arctica*	*Haliaeetus albicilla*	Czech Republic	Máca & Gonzalez-Solís (2022)	99.60	1032	99
28S gene							
KF601312	*S. arctica*	*Vulpes lagopus*	Noruega	Gjerde & Schulze (2014)	98.62	1528	100
KX022105	*S. arctica*	*Canis lupus*	USA	Calero-Bernal et al. (2016)	98.62	1497	100
KY947309	*S. arctica*	*Vulpes lagopus*	USA	Cerqueira-Cézar et al. (2017)	98.62	1502	100
MH469239	*S. caninum*	*Canis familiaris*	China	Ye et al. (2018)	98.62	3285	100

Roadkill wild animals represent an important source of research material. According to Pavlásek & Máca (2017), molecular methods are considered more sensitive in detecting protozoa such as *Sarcocystis* spp. Since no macro or microcysts were found, it remains unclear whether the sample size or the potential initial decomposition of tissues influenced the detection of sarcocysts.

Currently, there are no reports on *Sarcocystis* spp. in crab-eating raccoons (*P. cancrivorus*) worldwide. Previous research conducted in the states of São Paulo and Paraná, Brazil, also did not detect the presence of *Sarcocystis* spp. in roadkill *P. cancrivorus* (Richini-Pereira et al., 2016; Balbino et al., 2022). As previously mentioned, *Procyon* species are definitive and intermediate hosts of several other *Sarcocystis* species. However, it is still unclear whether *P. cancrivorus* are aberrant hosts of *Sarcocystis* or whether there is a regular cycle that involves transmission through their muscles, as no macro and microcysts were observed in the samples studied. Furthermore, the investigation of sarcocysts only in the cardiac musculature is an important limiting factor in our study, as other muscle groups (e.g., tongue, limbs) were not analyzed due to sample availability, which could increase the chances of identifying these structures.

Some authors have suggested that muscular sarcocystosis in carnivores results from accidental or unusual infections, due to immunosuppression (Hill et al., 1988). However, this hypothesis is not strongly supported, as even clinically healthy carnivores may harbor sarcocysts (Anderson et al., 1992). Recently, severe myositis in dogs was associated with infection by two *Sarcocystis* species, including *S. caninum* (Hagner et al., 2018). The definitive hosts for *S. arctica* and *S. caninum* remain unknown (Dubey et al., 2016). However, it has been suggested that *S. arctica* mainly uses canids as intermediate hosts and mammalian or avian carnivores (scavengers) as definitive hosts (Gjerde & Schulze, 2014; Máca & Gonzalez-Solis, 2022).

Wild terrestrial carnivores' role in transmitting infectious diseases requires continued study. The crab-eating raccoon has a great adaptation to various types of habitats and specifically in Brazil, it is found across all biomes (Wilson & Mittermeier, 2009). In countries where it exists, this animal is hunted for its fur, and according to Rosenthal (2021), hunters play a crucial role in the dissemination of pathogens such as protozoa from the genus *Sarcocystis*, including zoonotic species. Discarding the remains of carcasses in the environment or feeding viscera of slaughtered animals to domestic animals (e.g., dogs) can function as a transmission pathway, spreading the pathogen to various animal species in the vicinity.

This is the first record of molecular detection of *S. arctica* in the cardiac muscle of *P. cancrivorus*. This discovery warrants further investigation to determine whether this species should be considered a new intermediate host or an aberrant host of the protozoan. Finally, it is crucial to assess the impact of this parasite on *P. cancrivorus* populations.
